# Association between lipid profile changes and risk of in-stent restenosis in ischemic stroke patients with intracranial stenosis: A retrospective cohort study

**DOI:** 10.1371/journal.pone.0284749

**Published:** 2023-05-10

**Authors:** Jae-Chan Ryu, Jae-Han Bae, Sang Hee Ha, Boseong Kwon, Yunsun Song, Deok Hee Lee, Bum Joon Kim, Dong-Wha Kang, Sun U. Kwon, Jong S. Kim, Jun Young Chang

**Affiliations:** 1 Department of Neurology, Asan Medical Center, University of Ulsan College of Medicine, Seoul, Korea; 2 Department of Radiology, Asan Medical Center, University of Ulsan College of Medicine, Seoul, Korea; Foshan Sanshui District People’s Hospital, CHINA

## Abstract

**Objective:**

The risk of ischemic stroke with intracranial stenosis is associated with various serum lipid levels. However, the effects of changes in the lipid profile on the risk of in-stent restenosis have not been verified. Therefore, we investigated the association between the occurrence of in-stent restenosis at 12-month follow-up and changes in various lipid profiles.

**Methods:**

In this retrospective cohort study, we included ischemic stroke patients who had undergone intracranial stenting for symptomatic intracranial stenosis between February 2010 and May 2020. We collected data about serum low-density lipoprotein cholesterol (LDL-C), high-density lipoprotein cholesterol (HDL-C), total cholesterol (TC), and triglyceride (TG) levels, and calculated the TC/HDL-C and LDL-C/HDL-C ratios at baseline and after 12 months. We conducted multivariable logistic regression analyses to verify the association between various lipid profile changes and in-stent restenosis at 12 months.

**Results:**

Among the 100 patients included in the study (mean age, 60.8 ± 10.0 years; male: 80 [80.0%]), in-stent restenosis was found in 13 (13.0%) patients. The risk of in-stent restenosis of more than 50% was significantly decreased when TC/HDL-C ratio (odds ratio [OR] 0.22, [95% confidence interval (CI) 0.05–0.87]) and LDL-C/HDL-C ratio (OR 0.23, [95% CI 0.06–0.93]) decreased or when HDL-C levels (OR 0.10, [95% CI 0.02–0.63]) were increased at 12 months compared with baseline measurements.

**Conclusions:**

Improvement of HDL-C levels, TC/HDL-C ratio, and LDL-C/HDL-C ratio were associated with decreased risk of in-stent restenosis at 12-month follow-up. Management and careful monitoring of various lipid profiles including HDL-C levels, TC/HDL-C ratio, and LDL-C/HDL-C ratio may be important to prevent in-stent restenosis in patients with intracranial stenting.

## Introduction

Intracranial angioplasty and stenting can be considered for cases refractory to optimal medical treatment [1, 2]. The incidence of in-stent restenosis (ISR) is as high as 30% [3, 4]. Previous studies showed that lowering low-density lipoprotein cholesterol (LDL-C) levels may have a beneficial effect in patients with intracranial and extracranial artery stenosis [5, 6]. Also, statins reduced the risk of cardiovascular events in patients who underwent carotid revascularization [7].

Moreover, it was recently reported that various lipid profiles affect carotid artery restenosis after carotid endarterectomy or stenting [8]. Furthermore, other lipid profiles, such as the total cholesterol (TC)/LDL-C and LDL-C/high-density lipoprotein cholesterol (HDL-C) ratios, are associated with the risk of cardiovascular disease [9, 10]. However, only few studies have reported the effects of various lipid profiles on the prognosis of revascularization and ISR after intracranial stenting.

The purpose of the current study was to investigate the association between various lipid profiles including LDL-C and HDL-C levels, and TC/HDL-C and LDL-C/HDL-C ratios after statin treatment and the occurrence of ISR in patients who underwent intracranial stenting. We also investigated the association between the improvement of these lipid profiles and ISR after a 12-month follow-up.

## Methods

### Participants

We retrospectively reviewed the medical records of all patients with symptomatic intracranial stenosis who underwent intracranial stenting between February 2010 and May 2020 at Asan Medical Center (Seoul, South Korea). We included patients who fulfilled the following criteria: (1) age >18 years, (2) ischemic stroke or transient ischemic attack in the previous 6 months, and (3) stenosis of anterior circulation intracranial artery of at least 70%. We excluded patients who had a premorbid moderate-to-severe disability (modified Rankin scale score > 3). Demographic, vascular risk factors, and related laboratory data were obtained at baseline. This study was approved by the institutional review board of Asan Medical Center (IRB No. 2020–0726), which waived the need for written informed consent considering the retrospective nature of the study.

### Measurement of serum lipid profiles

We collected serum lipid samples at baseline and follow-up visits after 12 months. Serum lipid sampling was obtained after an overnight 12-hour fast because LDL-C, HDL-C, TC, and triglyceride (TG) levels are increased 3–6 hours after a meal. Based on the serum lipid measurements, we calculated the TC/HDL-C and LDL-C/HDL-C ratios. From this data, we dichotomized the lipid profiles according to whether these parameters improved. Improvement of TC, TG, and LDL-C levels, TC/HDL-C ratio, and LDL-C/HDL-C ratio was defined as a decrease at 12 months after baseline measurements, while improvement of HDL-C was defined as an increase at 12 months after baseline measurements.

High-intensity statin was defined as a reduction in LDL-C of >50% according to the 2013 American College of Cardiology/American Heart Association guideline. Administration of atorvastatin 40–80 mg or rosuvastatin 20–40 mg was classified as high-intensity statin therapy. Statin intensity was dichotomized into high intensity and low-to-moderate intensity.

### Stent procedure and follow-up intracranial neurovascular imaging

All patients received aspirin (100 mg/day) and clopidogrel (75 mg/day) for at least 7 days before procedure. The stenting procedure was performed under local anesthesia by experienced neuro-interventionists who chose the types of catheters, guidewires, and stents. Most intracranial stenting procedures were performed using the Wingspan^®^ (Boston Scientific Corporation, Fremont, CA, USA), Enterprise^®^ (Codman Neurovascular, Raynham, MA, USA), and Vision^®^ (Abbott Vascular, Santa Clara, CA, USA). After stent insertion, dual antiplatelet therapy was maintained at the discretion of the treating physician for at least 3–6 months [11].

Intracranial neurovascular imaging was performed using magnetic resonance angiography (MRA), computed tomography angiography (CTA), or digital subtraction angiography (DSA). Neurovascular imaging was performed on the day after intracranial stenting, and at the 12-month follow-up. The severity of intracranial artery stenosis before stent insertion was calculated by dividing the residual diameter by the vessel diameter at a point distal to the stenosis to obtain the normal vessel diameter. The severity of ISR was calculated by dividing the residual diameter at 12-month follow-up imaging by the residual stenosis measured right after stent insertion. According to the severity, ISR was defined as follows: mild (≤ 50%), moderate (50–70%), and severe stenosis or occlusion (70%–100%). The main outcome was the presence of more than 50% of restenosis in neurovascular imaging at 12-month follow-up after stent insertion. Outcomes for sensitivity analysis included the degree of ISR measured as an ordinal variable (mild, moderate, and severe stenosis or occlusion) and any degree of restenosis.

### Statistical analysis

The baseline characteristics of study patients were analyzed. Then, we stratified patients according to the presence of stenosis at follow-up neurovascular imaging and compared according to clinical characteristics and various serum lipid profiles and their changes (TC, TG, HDL-C, and LDL-C levels, and LDL-C/HDL-C and TC/HDL-C ratios). Continuous variables are presented as mean ± standard deviation (SD), while categorical variables are presented as frequency and percentage. The significance of intergroup differences was assessed using Pearson’s chi-square test, Fisher’s exact test, and Student’s t-test, as appropriate.

Improvement of serum lipid profiles was entered into multivariable binary and ordinal logistic regression analyses to identify the independent associations with the outcomes as described above. Model 1 was unadjusted. Model 2 was adjusted for age and sex. Model 3 was adjusted for age, sex, and statin intensity (low-to-moderate intensity vs. high intensity) considering that these factors are regarded to be clinically important. The odds ratios (ORs) are presented with 95% confidence intervals (CI). All analyses were performed using R (version 4.0.5; R Foundation for Statistical Computing, Vienna, Austria).

## Results

**Fig 1:**
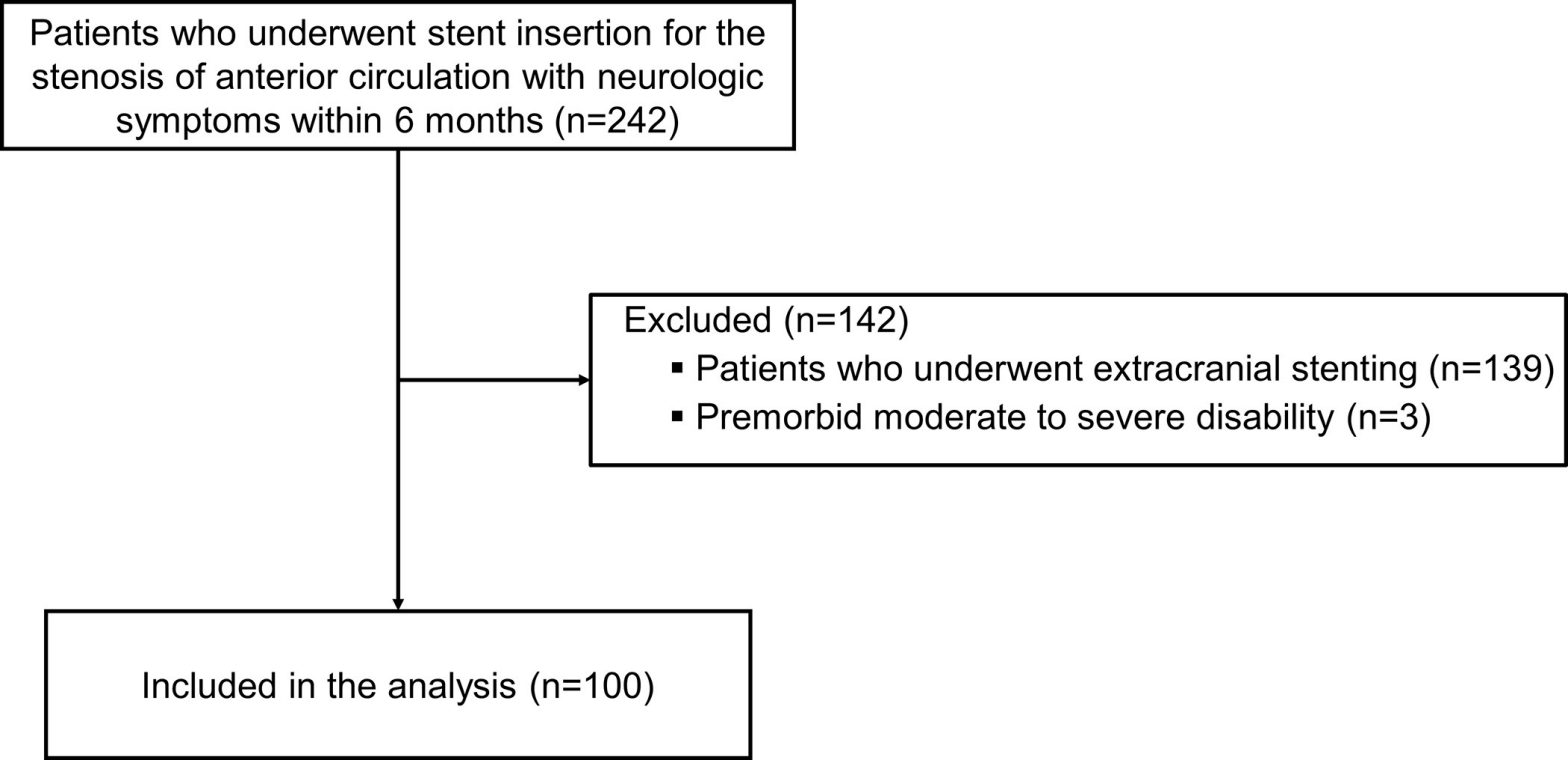
Flow diagram of this study.

During the study period, 100 patients underwent intracranial stenting for symptomatic severe stenosis refractory to medical treatment and were included in the analysis (Fig 1). Before stent insertion, all patients underwent DSA to calculate the severity of intracranial artery stenosis. At the 12-month follow-up after stent insertion, most patients (72%) underwent CTA for neurovascular imaging, followed by MRA (18%) and DSA (10.0%).

### Baseline characteristics of patients with intracranial stent insertion

The clinical characteristics of the study patients are summarized in Table 1. The mean age was 60.8±10.0 years, and 80 (80.0%) were male. Of them, 50 (50.0%) patients were prescribed high-intensity statins. Neurovascular imaging at 12-month follow-up revealed ISR in 13 (13.0%) patients (mild: 2 [2.0%], moderate: 7 [7.0%], severe stenosis or occlusion: 4 [4.0%]). Most of the stents were inserted into the middle cerebral artery (56 [56.0%]) and distal internal carotid artery (43 [43.0%]). Among the 13 patients with ISR, five patients had acute neurological deterioration when ISR was found. Three patients had a recurrent acute ischemic stroke in the stented territory, and two patients had a transient ischemic attack related to the stented territory. The types of stents used in the procedure were: Wingspan^®^ (55.0%), Enterprise^®^ (17.0%), Vision^®^ (15.0%), and others (14.0%).

**Table 1:** Baseline characteristics of patients with intracranial stent insertion.

Variable	Total (N = 100)
Age (mean ± SD), years	60.8 ± 10.0
Male sex, n (%)	80 (80.0)
Hypertension, n (%)	60 (60.0)
Diabetes, n (%)	32 (32.0)
Coronary artery disease, n (%)	8 (8.0)
Previous stroke, n (%)	31 (31.0)
Statin intensity, n (%)	
Low-to-moderate intensity	50 (50.0)
High intensity	50 (50.0)
Laboratory findings at admission (mean ± SD)	
TC, mg/dL	161.5 ± 45.1
TG, mg/dL	140.3 ± 94.0
LDL-C, mg/dL	100.8 ± 41.5
HDL-C, mg/dL	45.4 ± 16.4
TC/HDL-C ratio	3.9 ± 1.5
LDL-C/HDL-C ratio	2.5 ± 1.3
Laboratory findings at 12-month follow-up (mean ± SD)	
TC, mg/dL	137.1 ± 27.1
TG, mg/dL	132.8 ± 73.2
LDL-C, mg/dL	80.0 ± 21.9
HDL-C, mg/dL	45.4 ± 12.1
TC/HDL-C ratio	3.2 ± 0.8
LDL-C /HDL-C ratio	1.9 ± 0.7
Brain imaging at 12-month follow-up, n (%)	
Total ISR	13 (13.0)
Mild stenosis (0–50%)	2 (2.0)
Moderate stenosis (50–70%)	7 (7.0)
Severe stenosis or occlusion (70–100%)	4 (4.0)
Stenting location, n (%)	
MCA	56 (56.0)
Distal ICA	43 (43.0)
ACA	1 (1.0)
Types of stents, n (%)	
Wingspan^®^ stent	55 (55.0)
Enterprise^®^ stent	17 (17.0)
Vision^®^ stent	15 (15.0)
Others	14 (14.0)

SD, standard deviation; TC, total cholesterol; TG, triglycerides; HDL-C, high-density lipoprotein cholesterol; LDL-C, low-density lipoprotein cholesterol; ISR, in-stent restenosis; MCA, middle cerebral artery; ICA, internal carotid artery; VA, vertebral artery; BA, basilar artery; ACA, anterior cerebral artery.

### Baseline characteristics according to the presence of ISR > 50%

The comparison of clinical characteristics is summarized according to the presence of ISR > 50% (Table 2). Except for the younger mean age in the ISR > 50% group (61.8 ± 9.7 years vs. 53.5 ± 10.1 years, P = 0.009), there were no significant differences in sex, vascular risk factors, and statin intensity between the two groups. The LDL-C levels, TC/HDL-C ratios, and LDL-C/HDL-C ratios were significantly higher at baseline in patients with ISR ≤ 50% (P = 0.032, P = 0.046, and P = 0.036, respectively). There were no significant differences in the improvement of various lipid profiles between the two groups.

**Table 2:** Comparison of baseline characteristics stratified by the occurrence of ISR > 50%.

Variable	ISR ≤ 50% (N = 89)	ISR > 50% (N = 11)	P-value
Age (mean ± SD), years	61.8 ± 9.7	53.5 ± 10.1	0.009*
Male sex, n (%)	70 (78.7)	10 (90.9)	0.576
Hypertension, n (%)	52 (58.4)	8 (72.7)	0.557
Diabetes, n (%)	28 (31.5)	4 (36.4)	>0.999
CAD, n (%)	7 (7.9)	1 (9.1)	>0.999
Previous stroke, n (%)	26 (29.2)	5 (45.5)	0.451
Statin intensity, n (%)			0.523
Low-to-moderate intensity	43 (48.3)	7 (63.6)	
High intensity	46 (51.7)	4 (36.4)	
Laboratory findings at baseline (mean ± SD)			
TC, mg/dL	164.2 ± 46.1	139.1 ± 29.5	0.081
TG, mg/dL	143.2 ± 98.6	116.5 ± 35.1	0.082
LDL-C, mg/dL	103.9 ± 41.8	75.6 ± 30.0	0.032*
HDL-C, mg/dL	44.9 ± 16.7	49.2 ± 14.2	0.418
TC/HDL-C ratio	4.0 ± 1.5	3.0 ± 1.1	0.046*
LDL-C /HDL-C ratio	2.6 ± 1.3	1.7 ± 1.0	0.036*
Laboratory findings at 12-month follow-up (mean ± SD)			
TC	138.3 ± 27.4	127.1 ± 23.2	0.198
TG	133.7 ± 74.4	125.6 ± 66.0	0.731
LDL-C	80.6 ± 22.5	74.5 ± 15.1	0.386
HDL-C	45.2 ± 11.8	46.7 ± 15.1	0.694
TC/HDL-C ratio	3.2 ± 0.9	2.9 ± 0.6	0.191
LDL-C/HDL-C ratio	1.9 ± 0.7	1.7 ± 0.5	0.337
Improvement between admission and 12-month follow-up, n (%)			
TC↓	61 (68.5)	9 (81.8)	0.577
TG ↓	43 (48.3)	6 (54.5)	0.944
LDL-C ↓	61 (68.5)	5 (45.5)	0.235
HDL-C ↑	50 (56.2)	3 (27.3)	0.136
TC/HDL-C ratio ↓	63 (70.8)	4 (36.4)	0.051
LDL-C /HDL-C ratio ↓	62 (69.7)	4 (36.4)	0.063
Stenting location, n (%)			0.827
MCA	49 (55.1)	7 (63.6)	
Distal ICA	39 (43.8)	4 (36.4)	
ACA	1 (1.1)	0 (0.0)	

ISR, in-stent restenosis; SD, standard deviation; CAD, coronary artery disease; TC, total cholesterol; TG, triglycerides; HDL-C, high-density lipoprotein cholesterol; LDL-C, low-density lipoprotein cholesterol

* P < 0.05.

### Association between various serum lipid profiles after statin treatment and ISR

The associations between various outcomes and variables with the presence of ISR > 50% are summarized in Table 3. Improvements in HDL-C levels (OR 0.10 [95% CI, 0.02–0.63], P = 0.014), TC/HDL-C ratio (OR 0.22 [95% CI, 0.05–0.87], P = 0.031), and LDL-C/HDL-C ratio (OR 0.23 [95% CI, 0.06–0.93], P = 0.039) were significantly associated with the occurrence of ISR > 50% in Model 3 adjusted age, sex, and statin intensity.

**Table 3:** Binary and ordinal logistic regression analysis of predictors for the occurrence of ISR > 50%.

Outcome/Variables	Unadjusted: model 1		Adjusted: model 2		Adjusted: model 3	
	OR (95% CI)	P-value	OR (95% CI)	P-value	OR (95% CI)	P-value
≤ 50% ISR vs. > 50% ISR						
LDL-C ↓	0.38 (0.11–1.36)	0.138	0.46 (0.12–1.77)	0.261	0.44 (0.11–1.73)	0.241
HDL-C ↑	0.20 (0.04–0.97)	0.046*	0.10 (0.02–0.63)	0.014*	0.10 (0.02–0.63)	0.014*
TC/HDL-C ratio ↓	0.24 (0.06–0.87)	0.031*	0.23 (0.06–0.91)	0.037*	0.22 (0.05–0.87)	0.031*
LDL-C/HDL-C ratio ↓	0.25 (0.07–0.92)	0.037*	0.25 (0.06–0.98)	0.047*	0.23 (0.06–0.93)	0.039*
ISR (-) vs. ISR (+)						
LDL-C ↓	0.39 (0.12–1.26)	0.114	0.54 (0.15–1.90)	0.337	0.51 (0.14–1.83)	0.300
HDL-C ↑	0.27 (0.07–1.04)	0.057	0.17 (0.04–0.77)	0.022*	0.16 (0.03–0.76)	0.021*
TC/HDL-C ratio ↓	0.37 (0.11–1.19)	0.095	0.38 (0.11–1.34)	0.132	0.34 (0.09–1.22)	0.098
LDL-C/HDL-C ratio ↓	0.27 (0.08–0.89)	0.032*	0.30 (0.08–1.06)	0.062	0.26 (0.07–0.96)	0.043*
ISR (mild, moderate, severe stenosis or occlusion)^†^						
LDL-C level ↓	0.41 (0.13–1.33)	0.136	0.57 (0.16–1.99)	0.379	0.54 (0.15–1.92)	0.338
HDL-C level ↑	0.25 (0.07–0.99)	0.049*	0.18 (0.04–0.79)	0.023*	0.17 (0.04–0.78)	0.023*
TC/HDL-C ratio ↓	0.37 (0.11–1.21)	0.101	0.40 (0.12–1.38)	0.147	0.36 (0.10–1.26)	0.108
LDL-C/HDL-C ratio ↓	0.27 (0.08–0.91)	0.034*	0.32 (0.09–1.13)	0.077	0.28 (0.08–1.03)	0.055

Model 1: unadjusted, Model 2: adjusted for age and sex, Model 3: adjusted for age, sex, and statin intensity.

OR, odds ratio; CI, confidence interval; ISR, in-stent restenosis; LDL-C, low-density lipoprotein cholesterol; HDL-C, high-density lipoprotein cholesterol; TC, total cholesterol.

* P < 0.05

† Ordinal logistic regression analysis was performed.

When the outcome was dichotomized according to the occurrence of ISR, the association with the improvement of HDL-C levels (OR 0.16 [95% CI, 0.03–0.76], P = 0.021) and LDL-C/HDL-C ratio was maintained (OR 0.26 [95% CI, 0.07–0.96], P = 0.043). Ordinal logistic regression analyses also showed a consistent trend for the improvement in HDL-C levels (OR 0.17 [95% CI, 0.04–0.78], P = 0.023) and the LDL-C/HDL-C ratio (OR 0.28 [95% CI, 0.08–1.03], P = 0.055).

Additionally, we investigated the association between the discordance of LDL-C levels and the occurrence of ISR. Of the 50 patients with LDL-C levels below the median LDL-C level, which were regarded as being well-controlled, 43 patients had lower non-HDL-C levels (concordant) and the remaining seven patients had higher non-HDL-C levels (discordant). ISR was observed in eight patients (18.6%) in the concordant group and in two patients (28.6%) in the discordant group (P = 0.481) (S1 Table).

## Discussion

This study showed that improvements in HDL-C levels and TC/HDL-C and LDL-C/HDL-C ratios decreased the risk of the occurrence of ISR > 50% at 12-month follow-up. The association was maintained when the outcome was changed to the occurrence of ISR or analyzed as an ordinal variable (mild, moderate, and severe degree of restenosis). Unlike previous studies that investigated lipid measurements at one time point, we have longitudinally measured and analyzed the changes in various lipid profiles between baseline and at 12-month follow-up.

In previous studies, baseline lipid parameters were shown to be positively associated with cardiovascular disease [12–14]. Contrary to these prior studies, baseline LDL-C levels and TC/HDL-C and LDL-C/HDL-C ratios were significantly lower in the restenosis group in the current study. This may be due to the fact that patients without ISR had higher LDL-C levels before the administration of statin than those with ISR. Therefore, more patients without ISR received high-intensity statin within the preprocedural period. After 12 months of statin treatment, there was no significant difference in LDL-C levels between the two groups, suggesting that using high-intensity statins during a periprocedural period not only reduces the risk of periprocedural stroke but may also reduce the long-term risk of ISR.

ISR results from neo-intimal hyperplasia and early-stage vascular remodeling [15]. Proliferation and migration of vascular smooth muscle cells were suggested as the main causes for neo-intimal hyperplasia after stent insertion [16]. Also, elevated high-sensitive C-reactive protein, one of the biomarkers in systemic inflammation, was associated with ISR in coronary intervention [17]. Statins exert a diverse range of pleiotropic effects against these restenosis cascades, such as the inhibition of vascular smooth muscle cell proliferation, improvement of endothelial dysfunction, stabilization of atherosclerotic plaques, inhibition of platelet aggregation and intravascular inflammation, and enhanced anti-oxidative processes [18, 19]. Thus, using high-intensity statin during a periprocedural period may be as important as using antiplatelet drugs for preventing ISR.

Recently, there has been growing interest in the predictive role of lipid profiles on cardiovascular outcomes. Atherogenic lipoprotein particle number or concentration measured via nuclear magnetic resonance is more closely related to cardiovascular risk than LDL-C levels. Similar results have been described with standard lipid panels including the TC/HDL-C ratio, apolipoprotein B100 levels, and non-HDL-C levels [20]. The residual cardiovascular risk after statin therapy is more strongly associated with non-HDL-C than with LDL-C or apolipoprotein-B levels [21]. Previous studies showed that the LDL-C/HDL-C ratio is a better predictor for ischemic stroke events or outcomes [22]. Although the mechanism of improved prediction remains to be verified, several explanations are possible. It is known that intracranial arteries have a higher antioxidant capacity and are more prone to oxidative stress than extracranial arteries [23]. HDL-C inhibits the oxidation of phospholipids and the oxidant activity of LDL-C, and low HDL-C levels may increase oxidative stress to a higher degree in intracranial arteries than in extracranial arteries [24]. Intracranial artery stenosis is associated with insulin resistance. Insulin resistance is related to higher fasting TG level and low HDL-C level [25, 26]. Therefore, including HDL-C or non-HDL-C beyond LDL-C may be more sensitive for predicting ISR.

Beyond various lipid profiles, we analyzed the effects of discordance of LDL-C. The prevalence of discordance between LDL-C and other lipid parameters ranges from 11% to 25%, and the cardiovascular risk may be over- or under-estimated in such patients. Although the risk of ISR was higher in patients with discordance between LDL-C and non-HDL-C, there was no statistical significance between the two groups due to the small number of patients.

Our study has several limitations. First, because this study was conducted only in a single race with a small sample size, the generalizability is limited even if we adjusted for some factors in multivariable models. Also, we could not perform a statistical power calculation because of the retrospective nature of this study as well as its main goal. These findings require further verification in a larger prospective study. Second, because lipid profiles were measured only twice, we could not calculate the variability parameters exactly. Third, although we adjusted for some clinical factors in multivariable logistic regression analysis, we could not consider other confounding factors such as food intake, dietary habit, and physical activity that can affect serum lipid profiles. Finally, we cannot exclude the possibility that some ISRs had already occurred before checking the lipid profiles at 12 months.

Despite these limitations, we have shown that improvements in various lipid profiles were associated with the occurrence of ISR at 12-month follow-up. Therefore, management and careful monitoring of various lipid profiles beyond LDL-C levels may be important to prevent ISR in patients with intracranial stenting.

## Supporting information

S1 Table Association between the discordance of LDL-C levels and the occurrence of ISR in patients with LDL-C levels below the median LDL-C level. (DOCX)
