# Supplementary material for: Association between lipid profile changes and risk of in-stent restenosis in ischemic stroke patients with intracranial stenosis: A retrospective cohort study
Source: PLoS One. 2023 May 10;18(5):e0284749. doi: 10.1371/journal.pone.0284749 (PMC10171672; doi:10.1371/journal.pone.0284749)
Supplement: S1 Table — (DOCX) [file pone.0284749.s001.docx]

**S1 Table. Association between the discordance of LDL-C levels and the occurrence of ISR in patients with LDL-C levels below the median LDL-C level**

|  | Concordant group with lower non-HDL-C levels (N=43) | Discordant group with higher non-HDL-C levels (N=7) | P-value |
| --- | --- | --- | --- |
| ISR | 8 (18.6) | 2 (28.6) | 0.481 |

LDL-C, low-density lipoprotein cholesterol; ISR, in-stent restenosis; HDL-C, high-density lipoprotein cholesterol.
